# Influence of Foliar Silicic Acid Application on Soybean (*Glycine max* L.) Varieties Grown across Two Distinct Rainfall Years

**DOI:** 10.3390/plants10061162

**Published:** 2021-06-08

**Authors:** Uppalige Shwethakumari, Thimmappa Pallavi, Nagabovanalli B. Prakash

**Affiliations:** Department of Soil Science and Agricultural Chemistry, University of Agricultural Sciences, Bangalore, Karnataka 560 065, India; me.shwetha94@gmail.com (U.S.); nagabovanalliprakash@rediffmail.com (N.B.P.)

**Keywords:** foliar application, silicon, yield, quality, nutrient uptake

## Abstract

The foliar nutrition of silicic acid is considered to be a novel approach in enhancing the performance of many crops worldwide. The present study aimed to assess if the foliar application of silicon (Si) could influence the performance of soybean varieties with distinct crop duration, MAUS-2 (long duration) and KBS-23 (short duration). Field experiments were conducted in two consecutive years (2016 and 2017) of varied rainfall with foliar application of silicic acid @ 2 and 4 mL L^−1^ for three and two sprays each. The results showed significant enhancement in the yield, seed quality (protein and oil content), and uptake of nutrients (N, P, K, Ca, Mg, S, and Si) by various parts *viz.*, seed, husk, and haulm of both varieties with foliar nutrition of silicic acid. However, the short duration variety, KBS-23, responded well under low rainfall conditions (2016) with two sprays of foliar silicic acid @ 4 mL L^−1^ and MAUS-2 variety in the second season under higher rainfall (2017) with three sprays of foliar silicic acid @ 2 mL L^−1^, along with the recommended dose of fertilizer. This research revealed that the effectiveness of foliar silicic acid nutrition differs with the duration of the varieties, number of sprays given, and water availability in the soil during the cropping period.

## 1. Introduction

Being the world’s most important seed legume, soybean (*Glycine max* L. Merril) contributes 25 per cent to the global oil consumption and about two-third of the world’s supply of protein. India constitutes 10 per cent of the world soybean area, but its contribution to total world soybean grain is only 4 per cent, indicating the poor levels of productivity of the crop in the country (1.1 t ha^−1^), as compared to other countries (world average 2.2 t ha^−1^) [1]. The demand for increased production of soybean is forecasted to mirror the world’s population growth and demand for protein and edible oil. The increasing gap between the demand and production certainly highlights the need to increase soybean production. Unpredictable weather, diseases, pests, weeds and variable soil quality, and improper use of varieties [2,3,4] are the main challenges and threats to soybean production.

Adequate soil fertilization with N, P, K, and other micronutrients is highly required in soybean production as it can affect physiological processes, which, in turn, influence grain yield and quality of soybean crop [5]. Apart from soil application, foliar spray of nutrients has been proven to be a practical means of replenishing the reservoir of nutrients in the leaves of legumes during pod development, since the efficiency of nutrient uptake by roots as well as symbiotic fixation activities are known to decline at this stage [6]. However, inclusion of beneficial elements like Si, along with the essential nutrients, would provide a balanced nutrition to the plants, and help in augmenting the production and productivity of the soybean crop.

Silicon (Si), the second-most abundant element in the earth’s crust after oxygen, accounts for about 28% of the soil weight [7]. Despite its abundance in the soil, Si is sometimes deficient as a nutrient element because it exists mostly as SiO_2_, a form that is not available for plant uptake. To be taken up by plants, Si must be in the form of monosilicic acid (H_4_SiO_4_); but the natural release of H_4_SiO_4_ from SiO_2_ is a very slow process. Silicon is mostly present in soil solution as silicic acid at concentrations of 0.1–0.6 mM [8,9]. However, Si is present in varying amounts in all terrestrial plants, ranging from 0.1 to 10 per cent of shoot dry weight [7,10,11]. This difference of Si levels in different plant species have been attributed to the Si uptake ability of the roots [12,13,14]. While its essentiality as a plant nutrient is still debated, there are many reports of plant response to the addition of Si through different sources of foliar and soil application—calcium silicate, diatomaceous earth, rice husk biochar, potassium silicate, silicic acid etc.—in different crops [15,16,17,18].

Recent studies on supplying Si through the leaves and on viable fertilization alternatives revealed promising response in various crops. Foliar application of silicon has been shown to (1) influence plant growth, yield and silicon content of different crops such as rice [19,20,21,22,23,24,25], finger millet [26], wheat [27,28,29], maize [30], soybean [31] sugar beet [32], banana [33] and tomato [34] (2) control disease in rice [35,36,37], soybean [38,39,40,41], grape [42], coffee [43], cucumber [44], and tomato [45]; and (3) induce resistance to abiotic stress in crops like rice [46,47], wheat [27,48,49,50], potato [51], and soybean [52].

Climate change, including rising temperatures and increasingly severe droughts, will hamper crop development and yields. Water deficit hinders plant growth and development, thereby causing compromised reproductive processes, restricted nutrient uptake by roots and their transport to shoots, which ultimately affect the growth and yield of the crop. Several studies have demonstrated the role of Si to alleviate diverse abiotic stresses (drought and low temperature) in plants through physiological, biochemical, and physical mechanisms, but the molecular aspect is still indistinct [50,51,53]. Foliar application of silicon has a biostimulative effect and the best results are observed in stressful conditions for plants such as salinity, deficiency or excess of water, high and low temperature, and the strong pressure of diseases and pests [49,54].

However, the effect of foliar application of Si on performance of soybean is majorly focused on its role in plant protection so far and its influence on yield, quality, and nutrient uptake of the crop has remained understudied, which is vital in achieving future global needs. In this context, the present investigation was undertaken to evaluate the effect of soluble silicic acid as foliar application on yield, seed quality, and nutrient uptake by soybean varieties in two varied rainfall years.

## 2. Results

### 2.1. Yield of Soybean

**Figure 1:**
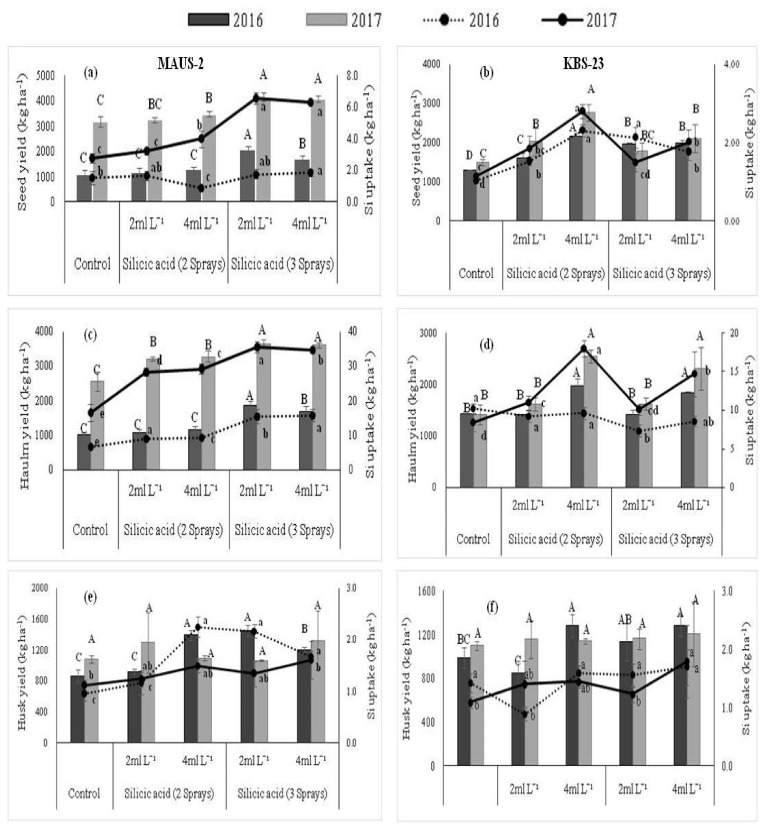
(**a***–***f**) Effect of foliar application of silicic acid on seed, haulm, and husk yield and Si uptake by short (KBS-23) and long duration (MAUS-2) soybean varieties in two distinct rainfall years. Means within a graph followed by the same capital and small letters are not significantly different at 5% level of significance for yield and uptake, respectively.

The foliar applied silicic acid had a significant effect on seed, haulm, and husk yield of soybean. A significant variation with respect to yield was noticed with varieties, levels of silicic acid application, and also the rainfall received during the growing seasons (Figure 1a–f). The MAUS-2 variety recorded significantly higher seed (Figure 1a), haulm (Figure 1c), and husk yield (Figure 1e) with the application of silicic acid @ 2 mL L^−1^ for three sprays over other treatments in both the seasons, except husk yield in the second season (2017). The higher seed and haulm yield of 4156 kg ha^−1^ and 3651 kg ha^−1^, respectively, was recorded in the second season, which received higher rainfall over the yield recorded during the first season (2054 kg ha^−1^ and 1871 kg ha^−1^ of seed and haulm yield, respectively).

The short duration variety KBS-23 responded well to the foliar application of silicic acid @ 4 mL L^−1^ two times, irrespective of the seasons. This variety recorded significantly higher seed (2770 kg ha^−1^; Figure 1b) and haulm (2548 kg ha^−1^; Figure 1d) yield during the second season (2017) over the previous season, 2016 (seed and haulm yield of 2177 kg ha^−1^ and 1977 kg ha^−1^, respectively).

### 2.2. Quality Parameters of Soybean

#### 2.2.1. Oil Content and Oil Yield

Application of silicic acid @ 4 mL L^−1^ two times significantly increased the oil content of MAUS-2 variety in the first season, but had no significant effect in the second season. Whereas in the KBS-23 variety, higher oil content of 17.84 per cent and 19.63 per cent was observed during first and second season in control (water spray) and with foliar application of silicic acid @ 4 mL L^−1^ three times, respectively (Table 1).

Irrespective of the seasons, application of silicic acid significantly increased oil yield of both the varieties. In both seasons, foliar applied silicic acid @ 2 mL L^−1^ three times significantly increased the oil yield to an extent of 420.42 kg ha^−1^ in the first season and 821.82 kg ha^−1^ in the second season in the MAUS-2 variety. In the KBS-23 variety, significantly higher yield was observed with the foliar application of silicic acid @ 4mL L^−1^ twice (337.87 kg ha^−1^ in 2016 and 533.53 kg ha^−1^ in 2017).

**Figure 2:**
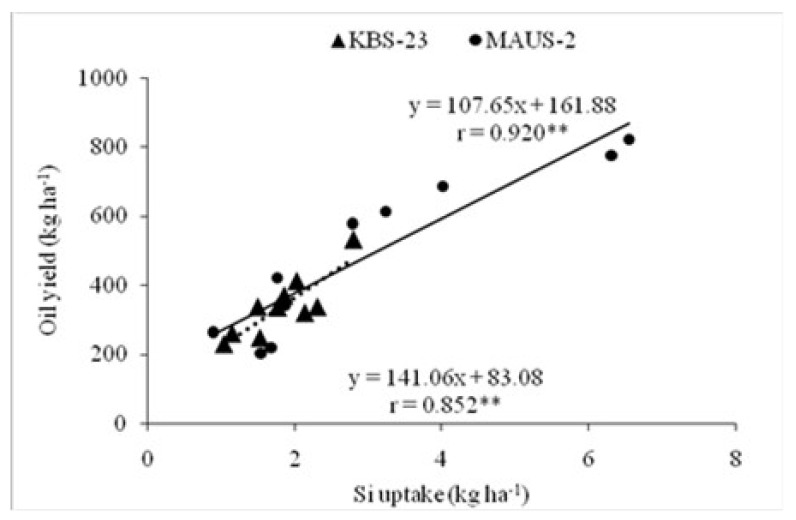
Relationship between Si uptake and oil yield of short (KBS-23) and long duration (MAUS-2) soybean varieties (pooled data of two years). (** The relationship is statistically significant at 0.01 level of significance).

Irrespective of the varieties, the oil yield showed a significant linear relationship with the Si uptake levels with correlation values of 0.920** and 0.852**, respectively, for the MAUS-2 and KBS-23 varieties (Figure 2).

#### 2.2.2. Protein Content and Protein Yield

**Figure 3:**
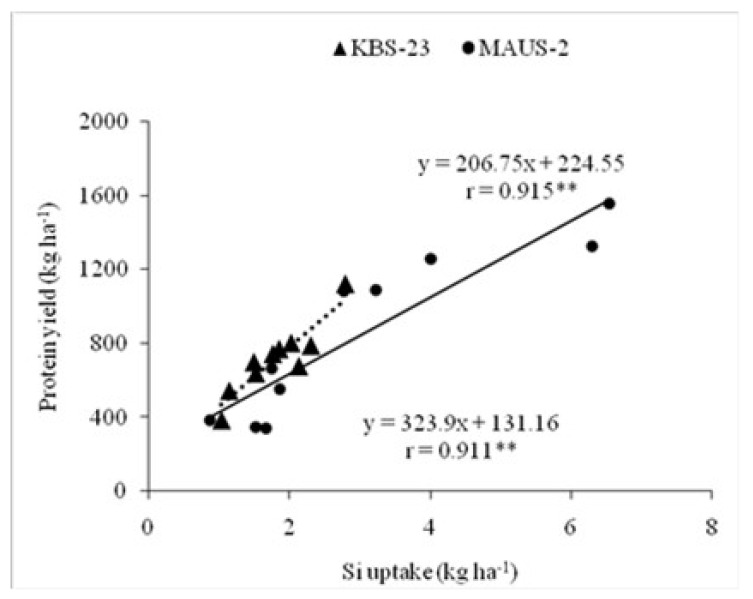
Relationship between Si uptake and protein yield of short (KBS-23) and long duration (MAUS-2) soybean varieties (pooled data of two years). (** The relationship is statistically significant at 0.01 level of significance).

The application of foliar silicic acid showed significantly enhanced protein content and protein yield in both the varieties (Table 1). However, comparatively higher protein content and yield were recorded in the second season over the first season, irrespective of the varieties. The MAUS-2 variety showed a significant response to foliar applied silicic acid @ 2 mL L^−1^ three times with protein content of 37.23 per cent and protein yield of 1547.51 kg ha^−1^ in the second season than the first season. Wherein, KBS-23 recorded significantly higher protein content (40.58%) and protein yield (1122.85 kg ha^−1^) with the foliar application of silicic acid @ 4 mL L^−1^ two times in the second than the first season. Both the varieties exhibited a significant relationship between their Si uptake levels and protein yield. Significant positive correlation coefficients of 0.915** and 0.911** were noticed with MAUS-2 and KBS-23, respectively (Figure 3).

### 2.3. Nutrient Uptake by Soybean

In MAUS-2, seed and haulm recorded significantly higher Si uptake of 1.88 kg ha^−1^ and 15.72 kg ha^−1^ in the first season than 6.55 kg ha^−1^ and 35.47 kg ha^−1^ in the second season with foliar silicic acid @ 4 mL L^−1^ thrice and 2 mL L^−1^ thrice, respectively over other treatments. Whereas, Si uptake by husk was significantly higher during first season (2.25 kg ha^−1^) over the second season (1.61 kg ha^−1^) with the foliar application of silicic acid @ 4 mL L^−1^ twice and thrice, respectively. In the KBS-23 variety, higher Si uptake by seeds, haulm, and husk was noticed in the second season than the first season, during which seeds and haulm recorded significantly higher Si uptake of 2.31 kg ha^−1^ and 9.62 kg ha^−1^ over 2.80 kg ha^−1^ and 17.98 kg ha^−1^, respectively, with foliar silicic acid @ 4 mL L^−1^ two times in both the seasons. However, Si uptake by husk was significantly higher with foliar silicic acid @ 4 mL L^−1^ three times in both the seasons (Figure 1a–f).

In both the seasons, the MAUS-2 variety recorded significantly higher N (106.59 and 247.60 kg ha^−1^), P (9.70 and 20.20 kg ha^−1^), and K (33.78 and 62.95 kg ha^−1^) uptake in 2016 and 2017, respectively) with the application of silicic acid @ 2 mL L^−1^ three times over control. While the KBS-23 variety recorded significantly higher uptake (131.19, 10.68, 34.76 kg ha^−1^ of N, P, and K, respectively, in 2016 and 179.66, 14.83, 38.88 kg ha^−1^ of N, P, and K, respectively in 2017) with the application of silicic acid @ 4 mL L^−1^ two times over the control (Table 2). It was also observed that application of foliar silicic acid enhanced the P and K uptake by husk and haulm of both the varieties during both seasons over the control (Table 2). In MAUS-2, foliar application of silicic acid @ 2 mL L^−1^ three times significantly enhanced uptake of Ca, Mg, and S by seed, haulm, and husk during both seasons (Table 3). However, twice higher nutrient uptake of secondary nutrients was noticed during the second season (2017) than the previous season (2016). However, the KBS-23 variety recorded significantly higher uptake of Ca, Mg, and S with the application of silicic acid @ 4 mL L^−1^ two times.

## 3. Discussion

### 3.1. Yield of Soybean

Application of foliar silicic acid influenced crop growth by providing Si directly to the foliage and thereby enhancing seed, haulm, and husk yield (Figure 1a–f). Si is known to stimulate physiological responses in dicotyledonous plants like soybean [55], which includes increased net photo-synthesis, transpiration rate, stomatal conductance, intercellular CO_2_, and light interception [7], in turn enhancing crop yields. In addition, Si deposited on the leaf surface forms a protective barrier against invasion of pests and diseases as well as prevention of water losses through transpiration, imparting drought resistance [56]. As a result, pest and disease infestations were negligible during the cropping season in the present investigation. These cumulative effects of Si on soybean might have contributed to enhanced soybean yield in the study.

Being a long duration variety, MAUS-2 responded well to foliar application of silicic acid for more number of times (@ 2 mL L^−1^ for thrice) than KBS-23. While, KBS-23 responded well with application of silicic acid @ 4 mL L^−1^ two times, and no further significant improvement was noticed with increased dose and number of times of silicic acid application. The major fact behind this is that the KBS-23 variety is of short duration and application of silicic acid at a higher concentration (@ 4 mL L^−1^) twice could be sufficient to enhance yield because of efficient partitioning of photosynthates and better utilization of absorbed nutrients. Wherein MAUS-2, being a long duration variety, could assimilate applied silicic acid better, even at lower doses, but at more number of applications than KBS-23.

Among the two varieties, higher yield was recorded in KBS-23 during the first season and MAUS-2 during the second season. The varied rainfall distribution during the cropping period and its coincidence with cropping stages (vegetative and reproductive) of soybean has mainly attributed to the yield difference. Although provided with protective irrigation, the crop was affected during critical growth stages due to increased aerial temperature in the first season but the sufficient amount of rainfall received during the second season might have enhanced crop growth and yield of crop [57]. The varieties with longer lifecycle are more prone to water stress compared to early duration crop varieties [58,59] as seen between MAUS-2 and KBS-23 varieties in the study. The reproductive stages are the most sensitive phenological stages to temperature extremes across all species and during this developmental stage, temperature extremes greatly affect production [57]. These effects are evident in an increased rate of senescence, which reduces the ability of the crop to fill the grain or fruit efficiently. Vegetative and reproductive stages of varieties coincide with the foliar applied silicic acid, which help in efficient partitioning of photosynthates and better utilization of absorbed nutrients, thereby resulting in a higher yield.

Among the two seasons, better crop yield, nutrient uptake and quality parameters were observed during the second season over the first, attributed to adequate amount of rainfall received during the second season and the first season experienced water stress due to low and uneven distribution of rainfall (Table 4). It is interesting to note that the first season showed comparatively higher per cent increase in yield of soybean despite water stress, which is because of the application of silicon as silicic acid, which can enhance the biomass of the crop under water stress. Concordantly, Hattori et al. [60] observed that application of Si significantly improved root and shoot dry weight of sorghum under drought stress conditions, but had no effect on dry matter production under wet conditions. This indicates the facilitation of root growth and the maintenance of the photosynthetic rate and stomatal conductance at a higher level compared with plants grown without silicon application. Similarly, Ali et al. [61] suggested that K-silicate had the potential to alleviate the negative effects of water stress on sugar beet yield grown in calcareous soils. In general, many reports claim that the application of Si augments crop growth through physiological, biochemical, and physical mechanisms [51,53].

### 3.2. Quality Parameters of Soybean

Irrespective of the seasons, MAUS-2 recorded higher oil and protein yield than KBS-23 (Table 1). Due to a higher seed yield, with the application of silicic acid @ 2 mL L^−1^ three times in MAUS-2 and 4 mL L^−1^ two times in KBS-23, higher oil and protein yield was observed in the respective varieties and application rates. Short duration variety (KBS-23) responded well to the applied silicic acid under slightly water stressed condition in the first season and the MAUS-2 variety responded well in the second season because of a higher and even distribution of rainfall (Table 4), and effective utilization of applied Si by the plants resulted in higher oil and protein yield in both the seasons. Si nutrition is known to improve total nitrogen and total sulphur content as well their uptake, in turn increasing N and S containing amino acids, which further accounts for enhanced protein and oil content/yield [31,62,63]. Similarly, Schwarz [64] reported that Si can influence cell wall components, such as pectic acid and protein. Similarly, increase in protein content was also noticed in wheat [48] and in paddy [20] with the application of sodium silicate and silicon aqueous solution, respectively. However, data pertaining to the beneficial effects of foliar applied silicon on quality of crop is limited and its mode of action is still unknown.

### 3.3. Nutrient Uptake by Soybean

#### 3.3.1. Si Uptake by Soybean

A significant increase in Si uptake was noticed with the application of silicic acid in seed, haulm, and husk, which ranged from 0.88 to 6.55 kg ha^−1^, 6.51 to 35.47 kg ha^−1^ and 0.88–2.25 kg ha^−1^, respectively, in both varieties and seasons. It is important that different plant parts do accumulate nutrients in varied amounts. Accordingly, rice has a high ability to absorb and accumulate Si, varying greatly among different organs of the plant [23,65,66,67]. Similarly, Sandhya et al. [26] reported greater variation in Si accumulation in plant parts of finger millet with the foliar application of silicic acid and highest Si accumulation was observed in the glumes, followed by straw and least in grains. Likewise, in our study, application of foliar silicic acid has shown a higher Si uptake in haulm, followed by seed and husk, irrespective of the varieties. The higher Si uptake by haulm might be due to translocation of Si to the shoots in a non-rejective way [10,68]. Nolla et al. [69] found that application of calcium silicate at rates ranging from 0 to 12 Mg ha^−1^ to a Si-deficient soil increased Si content in soybean leaf tissue from 0.34 to 0.55 per cent. Rodrigues et al. [41], while studying the effect of foliar application of potassium silicate (KSi) on soybean, found that the Si content in leaf tissue was on an average, 1.1 and 0.75 per cent for the field and greenhouse experiments, respectively.

Among the two seasons, significantly higher Si uptake was observed in the first season over the second. The higher biomass recorded in the varieties due to higher amounts of rainfall aided in the greater uptake of Si by soybean in the second season.

#### 3.3.2. Uptake of Major Nutrients by Soybean

The foliar application of silicic acid has been found to have a substantial effect on uptake of nutrients *viz.*, N, P, K, Ca, Mg, and S by the studied soybean varieties. However, the MAUS-2 variety showed significantly higher uptake of nutrients with foliar application of silicic acid @ 2 mL L^−1^ and KBS-23 @ 4 mL L^−1^ (Table 2 and Table 3). The increased content and uptake of these nutrients has been majorly attributed to the fact that the Si addition enhances the expression of Si transporters, which, in turn, influences the uptake and translocation of these nutrients. In agreement with our findings, the results from other studies using foliar applied Si showed significant increase in uptake of nutrients by the crop. Savant et al. [70] noticed a positive interaction between Si and N in rice for higher per cent Si and its uptake in straw as well as grain yield. Miyake and Takahashi [71] reported that the N content of leaves, stems, and roots of soybean was consistently higher when Si was provided. Li et al. [72] reported that Si application greatly increased the concentration of N and P in corn plants. An increased nitrogen uptake in salt-stressed barley plants was also recorded, possibly due to the Si-stimulated root activity and plant vigor [73]. Ma and Takahashi [74] opined that the addition of Si raised the optimum P level in rice. Zhang et al. [75] noticed increase in uptake of NPK by rice with the application of calcium silicate to soil. It has been reported that most of the absorbed K accumulates in the shoot and a little is transferred to rice grains [76].

Similarly, the application of Si also increased the uptake of Ca, Mg, and S in soybean. He and Wang [77] reported that the application of Si fertilizer could enhance the uptake of Ca and Mg in wheat. Similarly, Venkataraju [30] reported that the application of foliar silicic acid enhanced S uptake in maize. Irrespective of soybean varieties, better uptake was observed in the second season over the first as a result of higher crop biomass due to higher amounts of rainfall in the second season.

## 4. Materials and Methods

### 4.1. Study Location and Its Characteristics

The two-year field experiment was conducted at Zonal Agricultural Research Station (12°58′ N latitude 77°35′ E longitude), University of Agricultural Sciences, Bengaluru, Karnataka, India, during kharif 2016 and 2017, to study the effect of foliar silicic acid on yield, quality, and nutrient uptake by soybean under varied rainfall conditions. The soil at the site was sandy loam with 71.07 per cent sand, 15.21 per cent silt, and 13.72 per cent clay; pH of 5.26, organic carbon of 4.7g kg^−1^, EC of 0.08 dS m^−1^ (1:2.5 water), and with avail. N, P_2_O_5_ and K_2_O content of 156.80, 463.63 and 185.47 (kg ha^−1^), respectively. The plant available Si content as extracted by acetic acid (AA) and calcium chloride (CC) was low (AA Si—29.12 mg kg^−1^ and CC Si—25.92 mg kg^−1^).

### 4.2. Climatic Conditions during Growing Seasons

The data on total amount of rainfall during each growing season was obtained from AICRP on Agrometeorology, Meteorological Observation at GKVK, Bengaluru, Karnataka, and is depicted in Table 4. In 2017, the total amount of rainfall received during the growing season (August–November) was 750.80 mm and was 6.8 times higher than 2016 (110.40 mm). The average maximum and minimum temperatures during the growing season were 28.75 and 18.18 °C, and 27.73 and 19.03 °C during 2016 and 2017, respectively.

### 4.3. Trial Establishment and Experimental Design

Before sowing, the land was ploughed to get good tilth and converted into required sized plots and leveled. Farm yard manure @ 6.25 t ha^−1^ was applied 15 days prior to the land preparation. The amount of fertilizer required per plot was determined (recommended dose of fertilizer (RDF) at the rate of 25:60:25 kg of N:P_2_O_5_:K_2_O ha^−1^ as urea, single super phosphate and muriate of potash, respectively along with zinc sulphate (12.5 kg ha^−1^) and incorporated into the soil. Soybean seeds were sown at the rate of 62.5 kg ha^−1^ with 30 cm × 10 cm spacing in plots measuring 5 m × 3.6 m in the month of August in both the seasons. Two soybean varieties with distinct duration (i.e., long duration—MAUS-2 and short duration—KBS-23) were used in this study [31]. 

Five treatments (Table 5) were imposed for each variety with four replications in a randomized complete block design.

The source of Si used in this study was concentrated soluble silicic acid, obtained from ReXil Agro BV, Chennai, India, which contains 2 per cent Si as soluble H_4_SiO_4_ [31]. The treatment structure consisted of silicic acid spray @ 2 mL L^−1^ and 4 mL L^−1^ at an interval of 15 days (2 sprays and 3 sprays) in such a way that it coincides with the vegetative and reproductive stages of soybean. The control plots were sprayed with water alone as a check. The silicic acid was sprayed at 2 and 4 mL L^−1^ with a spray volume of 250 L ha^−1^ for the first and second spray and 500 L ha^−1^ for the third spray, using clean tap water with negligible Si content. Spraying was done with a backpack sprayer of 20 L capacity (AGRIMATE, AM 505E) in the early morning during less wind to avoid drifting of spray droplet to adjoining plots.

### 4.4. Harvest and Yield Measurements

The above ground biomass of all plants was manually harvested at 83 DAS (KBS-23) and 105 DAS (MAUS-2) separately from the net plot by cutting the stalks to ground level with sickle. Threshing was exercised after drying of the produce and pod weight and seed weight was recorded per plot.

### 4.5. Determination of Si and Other Nutrients in Plant Samples

#### 4.5.1. Collection and Preparation of Plant Samples

Five plants were collected in the R7 stage (when one normal pod on the main stem was mature in colour) of the crop [78]. The samples were thoroughly washed with deionised water and oven dried at 65 °C until they reached a constant weight. Further, they were separated into haulm, husk, and seed; cut to pieces; dried; powdered; and stored for nutrient analysis.

#### 4.5.2. Estimation of Nutrient Content in Plant Samples

Plant samples were analysed for N [79], P [80], K [81], Ca [81], Mg [81], S [82], and Si [83] content by adopting standard procedures. The uptake of these nutrients was obtained by multiplying the dry weight of respective plant parts with percentage of corresponding nutrients and expressed as kg ha^−1^.

#### 4.5.3. Estimation of Si in Plant Samples

Soybean seed, haulm, and husk samples were microwave digested [83] and estimated for Si content by using a UV visible spectrophotometer [10,84].

### 4.6. Protein and Oil Content

The protein content in the seeds was analyzed by estimating nitrogen content by the micro-Kjeldahl method [79], then multiplied by a factor of 6.25 and expressed in percentage. The oil content of soybean seeds was estimated by the Soxhlet method [31]. Protein yield and oil yield were worked out on the basis of seed protein and oil content, respectively, multiplied with seed yield of soybean and expressed in kg ha^−1^.

### 4.7. Statistical Analysis

The data obtained from field observations and chemical analysis of soil and plant samples in both years were subjected to statistical scrutiny using analysis of variance (ANOVA) to find out the influence of silicic acid treatments on the yield, quality, and nutrient uptake by soybean varieties and the effects were tested at 5 per cent level of significance [85]. Correlation study was done using SPSS 2.0 software to determine the relationship between Si uptake and quality parameters of soybean.

## 5. Conclusions

Soybean is considered a wonder crop due to high protein (40–43%) and oil content (20%) and hence forecasted to mirror the world’s demand for protein and edible oil. Our investigation aims at improving quality parameters in soybean (protein and oil content) through foliar application of silicic acid, and has proven the same, besides enhancing yield and nutrient uptake. Accordingly, in our study, application of foliar silicic acid @ 2 mL L^−1^ three times and 4 mL L^−1^ two times, along with the recommended dose of fertilizer, was found to be effective in long duration (MAUS-2) and short duration (KBS-23) varieties, respectively. Silicon nutrition thus emerges as a novel approach to exploit the immense potential of soybean crop to meet global needs.

## Figures and Tables

**Table 1:** Effect of foliar silicic acid application on protein and oil content and yield of short (KBS-23) and long duration (MAUS-2) soybean varieties in two distinct rainfall years.

Treatments	Oil Content (%)		Oil Yield (kg ha^−1^)		Protein Content (%)		Protein Yield (kg ha^−1^)	
	2016	2017	2016	2017	2016	2017	2016	2017
	MAUS-2							
T1:RDF (Control)	18.69b	18.17b	202.25d	578.62c	32.08a	34.00bc	348.67b	1081.1c
T2: RDF + SA@ 2 mL L^−1^ at 21 and 36 DAS	19.06b	18.80ab	218.70d	612.95bc	29.46b	33.25bc	341.30b	1085.56c
T3: RDF + SA@ 4 mL L^−1^ at 21 and 36 DAS	20.95a	19.60a	263.49c	686.28b	30.92a	35.79ab	388.40b	1253.75b
T4: RDF + SA@ 2 mL L^−1^ at 21, 36 and 51 DAS	20.46a	19.73a	420.42a	821.82a	32.38a	37.23a	666.2a	1547.51a
T5: RDF + SA@ 4 mL L^−1^ at 21, 36 and 51 DAS	20.17a	18.97ab	340.34b	776.74a	32.96a	32.27c	554.17a	1321.88b
SEm ±	0.32	0.39	12.59	28.37	1.01	0.9	36.71	49.00
CD at 0.05	0.94	1.23	36.36	92.54	3.15	2.93	119.71	159.79
	**KBS-23**							
T1:RDF (control)	17.84a	17.47c	229.51b	260.45d	30.04b	36.63c	386.39c	545.97c
T2: RDF + SA@ 2 mL L^−1^ at 21 and 36 DAS	15.34c	18.37b	247.9b	370.98b	39.67a	38.21bc	639.65b	771.6b
T3: RDF + SA@ 4 mL L^−1^ at 21 and 36 DAS	15.52cd	19.27a	337.87a	533.53a	37.63a	40.58a	789.73a	1122.85a
T4: RDF + SA@ 2 mL L^−1^ at 21, 36 and 51 DAS	16.39bc	18.87b	320.63a	337.79c	34.71b	39.08ab	678.16b	700.36b
T5: RDF + SA@ 4 mL L^−1^ at 21, 36 and 51 DAS	16.85b	19.63a	336.57a	412.96b	37.33a	38.25bc	745.05a	804.04b
SEm ±	0.32	0.17	12.59	18.18	1.46	0.68	20.45	36.61
CD at 0.05	0.94	0.58	36.36	59.29	4.78	2.22	66.68	119.39

RDF: Recommended dose of fertilizer; SA: Silicic acid; DAS: Days after sowing; Note: Means within a graph followed by the same letters are not significantly different at 5% level of significance for yield and uptake, respectively.

**Table 2:** Effect of foliar silicic acid application on nitrogen, phosphorus, and potassium uptake (kg ha^−1^) by the seed, husk, and haulm of short (KBS-23) and long duration (MAUS-2) soybean varieties in two distinct rainfall years.

Treatments	MAUS-2						KBS-23					
	N		P		K		N		P		K	
	2016	2017	2016	2017	2016	2017	2016	2017	2016	2017	2016	2017
	Seed											
T1:RDF (control)	55.79b	172.98c	5.53b	16.15b	18.20c	48.06c	61.82c	87.35c	6.73b	8.26d	21.47d	21.18c
T2: RDF + SA@ 2 ml L^−1^ at 21 and 36 DAS	54.61b	17369c	5.84b	14.78b	19.46c	49.71bc	102.34bc	123.46b	8.20b	11.11b	26.44cd	28.47bc
T3: RDF + SA@ 4 mL L^−1^ at 21 and 36 DAS	62.14b	200.60b	6.33b	16.27b	20.69c	52.43b	131.19a	179.66a	10.68a	14.83a	34.76a	38.88a
T4: RDF + SA@ 2 mL L^−1^ at 21, 36 and 51 DAS	106.59a	247.60b	9.70a	20.20a	33.78a	62.95a	108.50b	112.06b	9.94a	8.79c	31.61b	24.39b
T5: RDF + SA@ 4 mL L^−1^ at 21, 36 and 51 DAS	88.67a	211.50a	8.41a	21.63a	28.13b	60.35a	119.21a	128.65b	9.76ab	10.76bc	32.10b	29.05b
SEm ±	5.87	7.84	0.73	0.63	1.65	1.32	4.30	5.86	0.49	0.71	0.70	1.62
CD at 0.05	19.15	25.57	2.37	2.04	5.37	4.29	14.01	19.10	1.58	2.31	2.29	5.30
	**Husk**											
T1:RDF (control)	-	-	0.63b	0.80a	9.12b	9.22b	-	-	0.43b	1.04a	8.88a	13.64b
T2: RDF + SA@ 2 mL L^−1^ at 21 and 36 DAS	-	-	0.39b	1.06a	8.98b	13.58b	-	-	0.34b	0.98a	6.91a	14.56a
T3: RDF + SA@ 4 mL L^−1^ at 21 and 36 DAS	-	-	0.92a	0.80a	18.83a	10.77a	-	-	0.78ab	1.79a	15.01a	14.95a
T4: RDF + SA@ 2 mL L^−1^ at 21, 36 and 51 DAS	-	-	0.88ab	0.97a	13.83ab	9.93b	-	-	0.70ab	1.01a	10.42a	15.88a
T5: RDF + SA@ 4 mL L^−1^ at 21, 36 and 51 DAS	-	-	0.81ab	0.97a	14.70ab	14.68a	-	-	0.89a	1.15a	12.14a	15.97a
SEm ±	-	-	0.08	0.14	2.11	1.12	-	-	0.11	0.43	1.65	1.04
CD at 0.05	-	-	0.26	NS	6.88	3.64	-	-	0.37	NS	NS	2.38
	**Haulm**											
T1:RDF (control)	-	-	0.62b	2.75a	5.54b	13.97a	-	-	1.75ab	1.54b	7.83a	7.71b
T2: RDF + SA@ 2 mL L^−1^ at 21 and 36 DAS	-	-	1.13ab	3.49a	6.51b	20.15a	-	-	1.48b	1.70b	7.46a	7.51b
T3: RDF + SA@ 4 mL L^−1^ at 21 and 36 DAS	-	-	1.11ab	3.41a	5.67b	19.07a	-	-	2.12a	2.53a	8.62a	10.52a
T4: RDF + SA@ 2 mL L^−1^ at 21, 36 and 51 DAS	-	-	1.68a	3.79a	9.89a	20.18a	-	-	1.55b	1.72b	8.18a	7.62b
T5: RDF + SA@ 4 mL L^−1^ at 21, 36 and 51 DAS	-	-	1.61a	3.75a	9.68a	18.91a	-	-	1.14b	2.27a	9.10a	11.30a
SEm ±	-	-	0.20	0.11	0.75	1.96	-	-	0.14	0.15	0.92	0.77
CD at 0.05	-	-	0.66	NS	2.46	NS	-	-	0.46	0.50	NS	2.50

RDF: Recommended dose of fertilizer; SA: Silicic acid; DAS: Days after sowing; NS: Non-significant. Note: Means within a graph followed by the same letters are not significantly different at 5% level of significance for yield and uptake, respectively.

**Table 3:** Effect of foliar silicic acid application on calcium, magnesium, and sulphur uptake (kg ha^−1^) by the seed, husk, and haulm of short (KBS-23) and long duration (MAUS-2) soybean varieties in two distinct rainfall years.

Treatments	MAUS-2						KBS-23					
	Ca		Mg		S		Ca		Mg		S	
	2016	2017	2016	2017	2016	2017	2016	2017	2016	2017	2016	2017
	Seed											
T1:RDF (control)	4.54b	10.7e	3.303a	10.55b	4.05c	11.18b	5.10b	6.76d	5.065a	4.78b	1.88c	5.04b
T2: RDF + SA@ 2 mL L^−1^ at 21 and 36 DAS	5.45b	13.21d	3.503b	8.93b	4.97c	12.48ab	6.40b	9.39b	5.197a	6.37ab	5.95b	8.44a
T3: RDF + SA@ 4 mL L^−1^ at 21 and 36 DAS	5.01b	15.54c	2.923b	10.75b	4.22c	10.97b	8.16a	12.84a	5.880a	8.75a	9.15a	11.68a
T4: RDF + SA@ 2 mL L^−1^ at 21, 36 and 51 DAS	9.14a	20.42a	5.050a	14.24a	13.66a	14.18ab	8.19a	7.88c	5.027a	5.66ab	6.41b	6.86a
T5: RDF + SA@ 4 mL L^−1^ at 21, 36 and 51 DAS	6.37b	16.97b	4.903ab	12.12ab	9.60b	15.04a	8.16a	8.12c	6.750a	6.24ab	6.19b	8.85a
SEm ±	0.81	0.3	0.44	1.3	0.96	0.8	0.47	0.2	0.611	0.8	0.72	1.3
CD at 0.05	2.63	1.4	1.4	3.2	3.14	3.1	1.53	0.9	NS	3.1	2.33	6.4
	**Husk**											
T1:RDF (control)	12.54b	13.5a	6.96c	8.32b	0.60b	1.5a	9.69a	14.07a	8.384a	8.34b	1.00	1.59a
T2: RDF + SA@ 2 mL L^−1^ at 21 and 36 DAS	13.21b	15.85a	7.03c	8.86a	1.03b	2.01a	8.96a	12.36a	6.126b	10.28a	1.39	1.68a
T3: RDF + SA@ 4 mL L^−1^ at 21 and 36 DAS	16.88a	14.1a	10.48b	7.22bc	1.93a	1.57a	12.95a	13.58a	9.439a	9.56ab	2.43	1.5a
T4: RDF + SA@ 2 mL L^−1^ at 21, 36 and 51 DAS	19.40a	12.68a	13.12a	7.09c	2.01a	1.58a	11.52a	12.a9a	8.144a	10.18a	1.99	1.6a
T5: RDF + SA@ 4 mL L^−1^ at 21, 36 and 51 DAS	15.73b	16.09a	10.60b	9.97a	2.19a	1.83a	12.63a	14.16a	8.913a	10.04ab	2.43	1.54a
SEm ±	1.05	0.2	0.674	0.41	0.16	0.21	1.03	0.61	0.604	0.31	0.20	0.12
CD at 0.05	3.44	NS	2.197	1.2	0.51	NS	NS	NS	1.971	1.8	0.66	NS
	**Haulm**											
T1:RDF (control)	17.05c	34.96a	6.788b	19.63a	1.04b	5.00a	27.52a	23.17d	11.451a	10.85a	2.06b	2.35a
T2: RDF + SA@ 2 mL L^−1^ at 21 and 36 DAS	18.45c	38.84a	7.392b	23.84a	1.20b	5.79a	24.99a	27.03c	11.114a	12.47a	1.59b	1.17a
T3: RDF + SA@ 4 mL L^−1^ at 21 and 36 DAS	18.02c	47.27a	8.015b	24.36a	1.30b	5.59a	37.81a	43.93a	19.304a	19.28a	3.59a	4.2a
T4: RDF + SA@ 2 mL L^−1^ at 21, 36 and 51 DAS	33.44a	46.37a	14.092a	27.49a	2.75a	6.26a	24.74a	26.89c	16.376a	12.97a	3.32a	2.63a
T5: RDF + SA@ 4 mL L^−1^ at 21, 36 and 51 DAS	27.80b	45.51a	13.076a	25.63a	3.23a	6.04a	33.48a	39.29b	12.968a	17.48a	3.34a	3.62a
SEm ±	1.25	1.2	0.387	2.4	0.25	0.33	3.31	1	2.092	2.1	0.25	1.24
CD at 0.05	4.07	NS	1.263	NS	0.82	NS	NS	3.31	NS	NS	0.81	NS

RDF: Recommended dose of fertilizer; SA: Silicic acid; DAS: Days after sowing; NS: Non-significant. Note: Means within a graph followed by the same letters are not significantly different at 5% level of significance for yield and uptake, respectively.

**Table 4:** Mean air temperatures and rainfall during the soybean growing season; data for the period for 2016 and 2017, AICRP, on Agrometeorology, Meteorological Observation at GKVK, Bengaluru.

Month/Year	2016			2017		
	Temp. (°C)		Rainfall (mm)	Temp. (°C)		Rainfall (mm)
	Max.	Min.		Max.	Min.	
August	28.10	19.50	28.00	28.20	20.00	199.80
September	27.80	19.00	51.40	27.70	19.60	275.60
October	29.60	18.00	31.00	28.10	19.00	264.00
November	29.50	16.20	0.00	26.90	17.50	11.40
**Mean/Sum**	**28.75**	**18.18**	**110.40**	**27.73**	**19.03**	**750.80**

**Table 5:** Treatment details.

Sl. No	Treatments
1	T_1_: Recommended dose of fertilizer (RDF) + water spray (Control)
2	T_2_: RDF + Silicic acid @ 2 mL L^−1^ at 21 and 36 days after sowing (DAS)
3	T_3_: RDF + Silicic acid @ 4 mL L^−1^ at 21 and 36 DAS
4	T_4_: RDF + Silicic acid @ 2 mL L^−1^ at 21, 36 and 51 DAS
5	T_5_: RDF + Silicic acid @ 4 mL L^−1^ at 21, 36 and 51 DAS

## Data Availability

Not applicable.
